# Time Kill Curve PD Modelling Experiments Are Affected by Trailing MIC Endpoints: Refinement of MIC Determination for *S. pseudintermedius*

**DOI:** 10.1111/jvp.70033

**Published:** 2025-10-28

**Authors:** Andrew Mead, Ludovic Pelligand

**Affiliations:** ^1^ Comparative Biomedical Sciences The Royal Veterinary College London UK; ^2^ Clinical Services and Sciences The Royal Veterinary College London UK

**Keywords:** pharmacodynamics, pharmacokinetics, PK/PD modelling, sulphonamide, trimethoprim

## Abstract

Trailing endpoints are a recognised challenge in broth microdilution MIC testing, particularly for bacteriostatic agents such as trimethoprim (TMP) and sulphonamides. In this study, we applied a pharmacodynamically guided refinement to determine the MIC of the combination against *Staphylococcus pseudintermedius* ; we did not aim at redefining clinical susceptibility, but at refining MIC determination to better guide pharmacodynamic study design. By providing more reliable thresholds for growth suppression, this approach supports optimisation of PD modelling and may ultimately inform translational applications, such as dose prediction and reducing misclassification in PD contexts. Visual MICs were compared to those derived from log_10_ changes in CFU/mL over 24 h, using pharmacodynamic thresholds of +2.3 log_10_ (growth from standard inoculum of 5 × 10^5^ to ~10^8^ CFU/mL, corresponding to visible growth MIC) and 0 log_10_ change (stationary concentration). Across 10 clinical isolates, visual MICs often underestimated the concentration required to suppress growth by 2–4 fold (more than one dilution step), particularly for sulphonamides. TMP‐sulphonamide combinations at a 1:19 ratio showed reduced trailing and closer agreement between visual and count‐based MICs, reflecting enhanced bactericidal activity. Time‐kill curve experiments anchored on the log_10_ count‐based MIC provided a well‐distributed range of PD responses, capturing both suppression and killing more accurately than curves centred on visual MICs. This method supports more rational selection of concentrations for PD studies and may be especially valuable for slow‐acting or ratio‐sensitive combinations, and has translational value for sulphonamides, such as sulfamethoxazole, used in both human and veterinary medicine.

Minimum inhibitory concentration (MIC) testing is central to veterinary antimicrobial pharmacology, informing both clinical decisions and pharmacodynamic (PD) modelling. EUCAST/CLSI presents guidelines for the measurement of MIC (CLSI 2024; EUCAST 2024). These involve exposing a standard inoculum to a 2‐fold dilution series of antimicrobial concentrations. MIC determination is typically reproducible for bactericidal agents with clear inhibition of growth/pellet formation (Gajic et al. 2022). However, interpretation can be problematic when growth inhibition is gradual or incomplete, leading to trailing endpoints. This occurs frequently with antifolates like trimethoprim (TMP) and sulphonamides (S), which yield indistinct growth‐inhibition transitions. Trailing typically results in a gradual decline in turbidity over several dilutions, without a clear inhibitory well. EUCAST (2024) advises that trailing is expected with TMP/S, recommending that the MIC be recorded as the lowest concentration inhibiting ≥ 80% of growth compared to the drug‐free control. However, this method remains subjective and vulnerable to variability, potentially leading to 4–16‐fold differences in MICs, which compromises reproducibility and complicates PD study design.

The central problem is that trailing MIC endpoints introduce ambiguity in determining the precise concentration that suppresses growth. This can lead to under‐ or overestimation of the MIC by several dilution steps. For pharmacology and PK/PD modeling, accurate MIC determination is more than a technical microbiology issue; it directly influences how concentration‐response relationships are positioned and interpreted, the design of time‐kill experiments, and the derivation of PK/PD indices such as AUC/MIC or T>MIC (Toutain et al. 2021 as illustrated in Figure 1). In this context, trailing endpoints can propagate into biased pharmacological interpretations if not carefully addressed.

**FIGURE 1:**
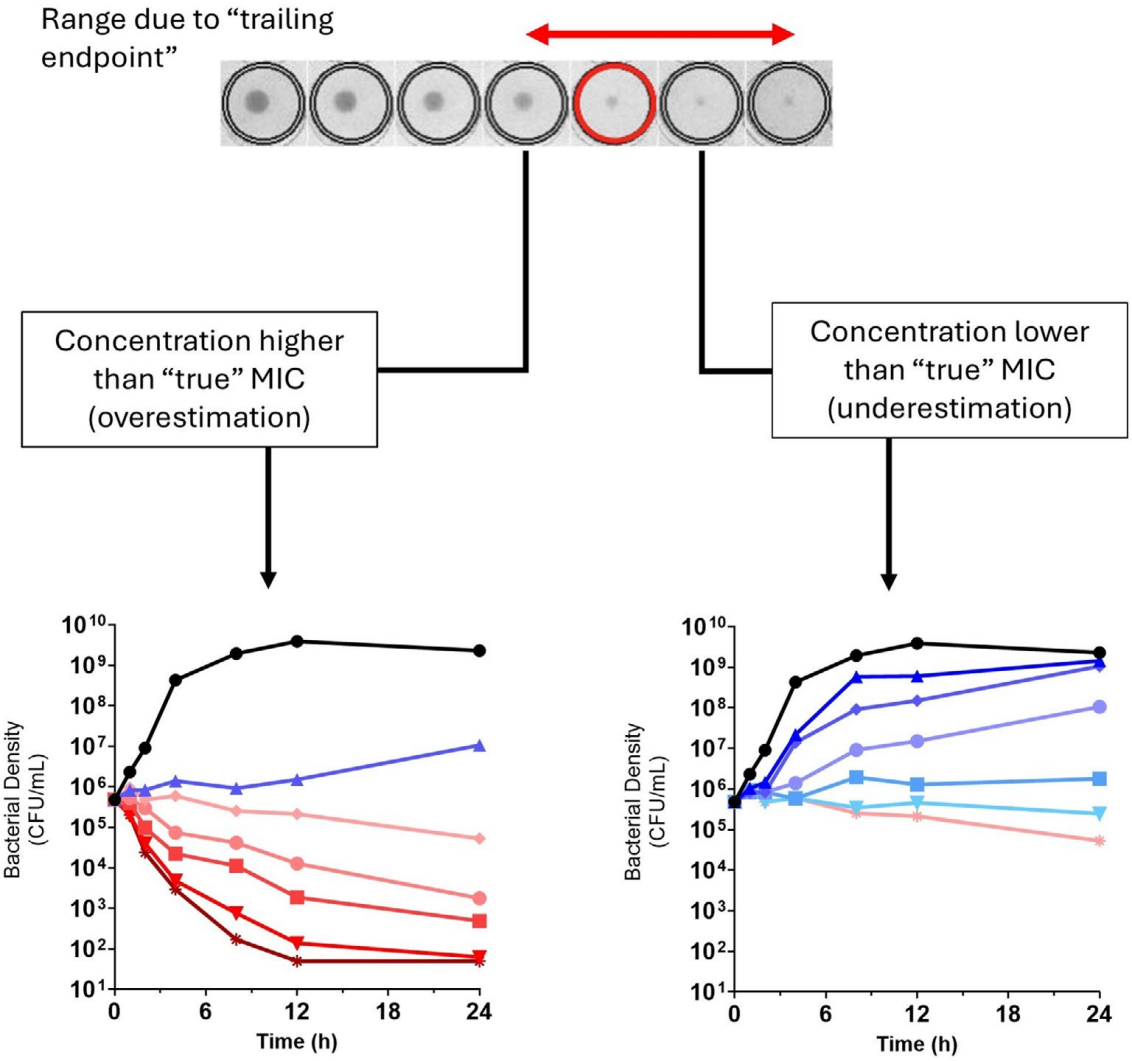
This representation highlights how trailing endpoints generate ambiguity and its potential impact on time‐kill curve (TKC) design for pharmacodynamic assessment of antifolates. Traditional MIC determination for trimethoprim and sulphonamides often results in trailing endpoints, with gradual turbidity reduction across 2–3 dilutions and no clear inhibitory concentration, making visual assignment of MIC subjective and inconsistent. This can lead to selection of suboptimal concentrations for TKC experiments, limiting the ability to capture relevant pharmacodynamic transitions (over estimation or underestimation of MIC scenarios).

A more mechanistic understanding of MIC emerges by linking it to bacterial density. Mouton and Vinks (2005) proposed that visual MIC corresponds to the concentration that limits growth to ~10^8^ CFU/mL, the threshold for visible turbidity/pellet. Given a standard inoculum of 5 × 10^5^ CFU/mL, this corresponds to a + 2.3 log_10_ increase in inoculum size, based on the difference between log_10_‐transformed values (1 × 10^8^ = 8.0; 5 × 10^5^ ≈ 5.7). In contrast, the stationary concentration (SC) is the drug concentration at which net bacterial growth is zero (0 log_10_ change). Importantly, these thresholds can be derived using pharmacodynamic parameters (EC_50_, E_max_ and slope) from time–kill curve (TKC) models (Nielsen and Friberg 2013).

While related, MIC and SC are not equivalent: MIC reflects the lower limit of visible growth, whereas SC denotes the biological threshold at which bacterial killing exactly offsets growth. In practice, this means that the MIC may lie one or more dilutions above the stationary concentration. Measuring bacterial density at the end of a standard MIC assay, particularly across dilutions spanning the visual MIC as exemplified in Figure 2, can therefore yield more precise and PD‐relevant data. This approach enhances TKC design by ensuring accurate anchoring of concentrations around key PD thresholds as visualised in Figure 3.

**FIGURE 2:**
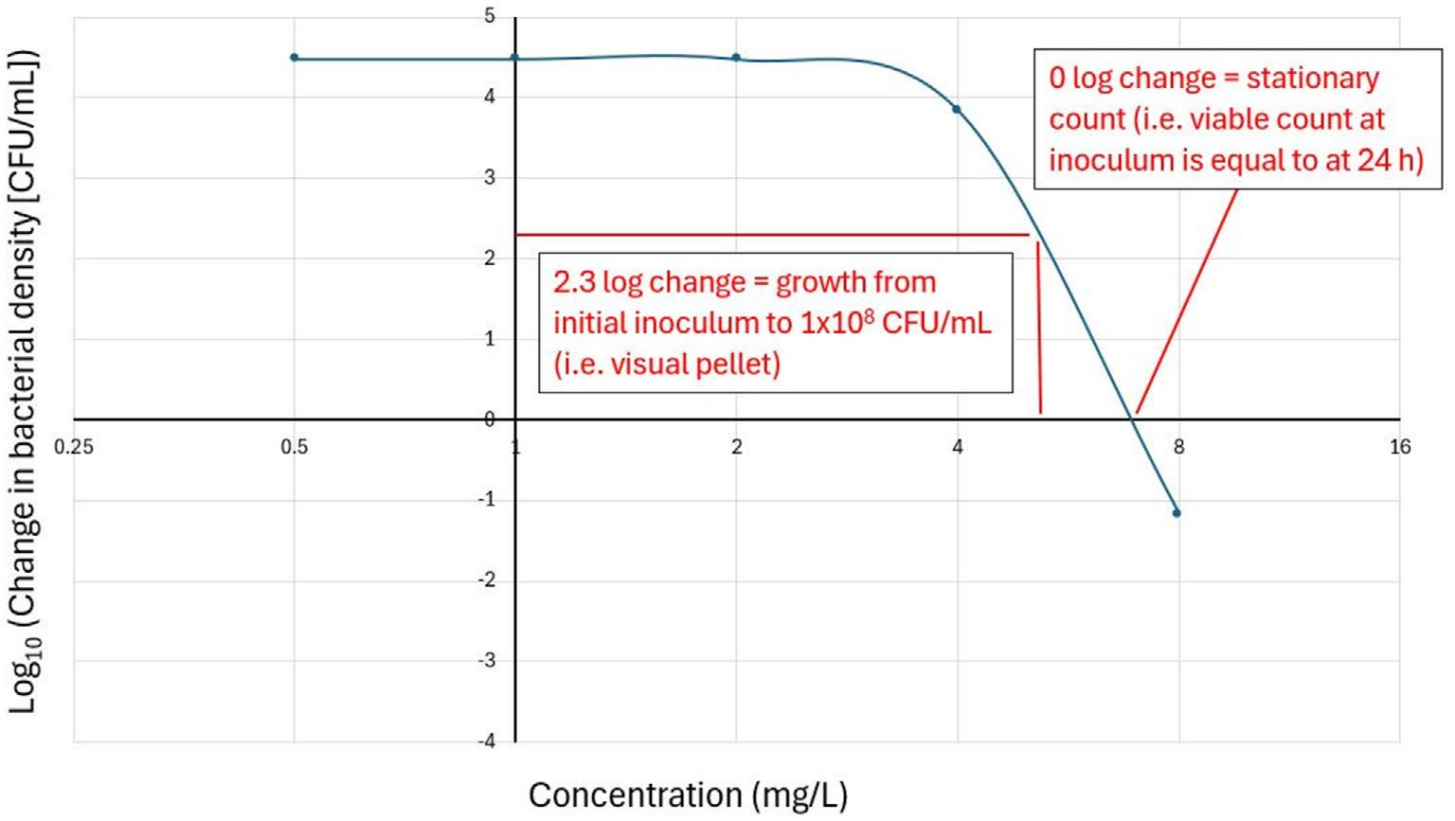
Representative example of the extended MIC determination method showing the change in bacterial density (log_10_ CFU/mL; *y*‐axis) after 24 h of incubation across increasing concentrations (mg/L; *x*‐axis) of an antifolate antimicrobial. The pharmacodynamic thresholds used for MIC assignment are shown: 0 log_10_ change represents stasis (no net growth from initial inoculum), and 2.3 log_10_ change corresponds to a bacterial density of 10^8^ CFU/mL (Mouton et al. 2005), typically associated with visible pellet formation. MIC by pharmacodynamic definition is the lowest concentration achieving stasis (Drusano 2004; Mouton and Vinks 2005).

**FIGURE 3:**
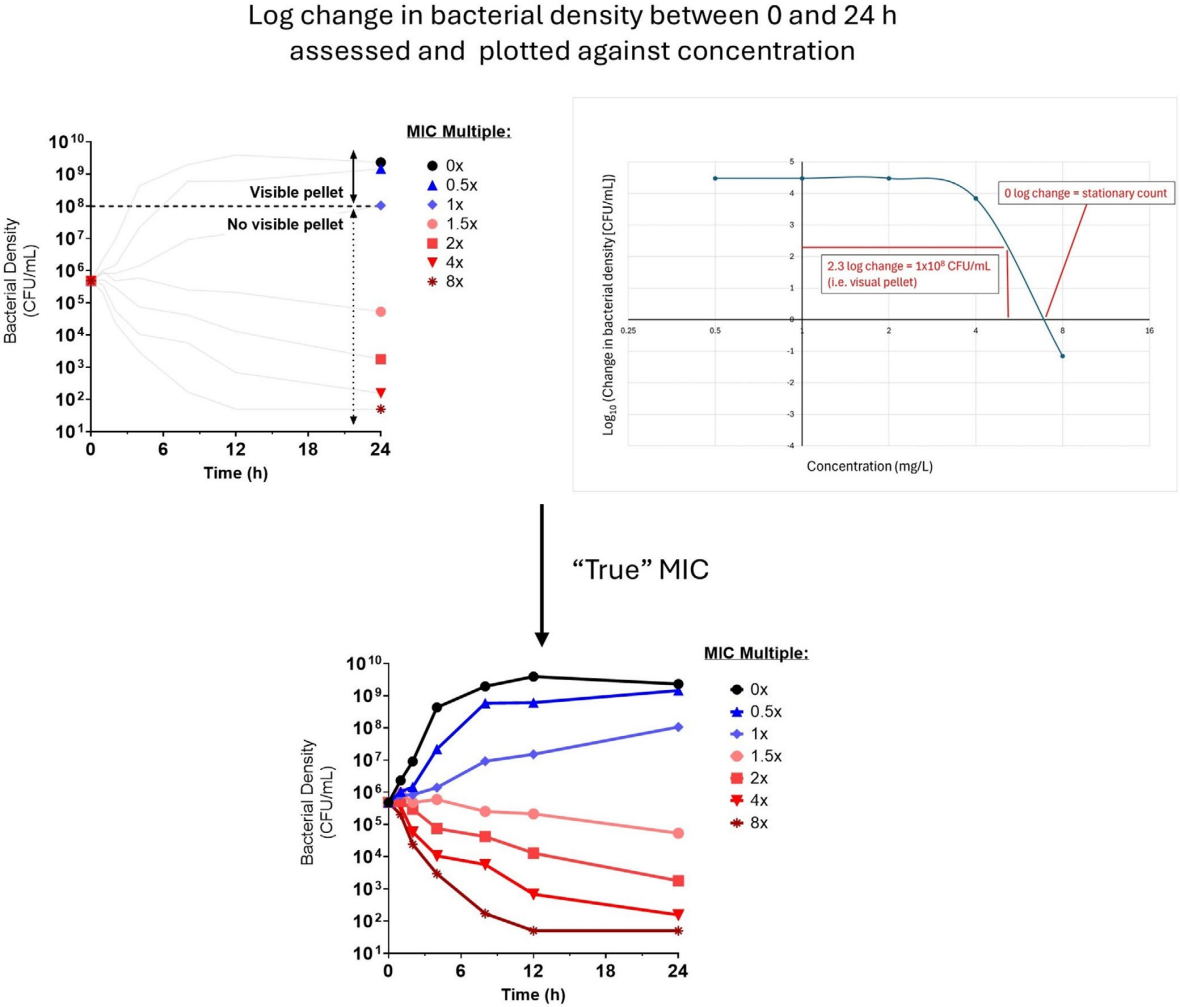
This representation indicates how bacterial density at the end‐point relates to MIC and can better centralise subsequent TKC. By quantifying bacterial counts at 24 h across wells spanning the visual MIC and plotting the log_10_ change from the initial inoculum to the growth threshold (~10^8^ CFU/mL), more precise stationary and growth thresholds can be identified resolving the uncertainty introduced by trailing. These refined endpoints support rational selection of TKC concentrations and improve the quality of PD data.

Conventionally, time–kill assays are employed to visualise dynamic antimicrobial activity and, in combination with checkerboard assays, to confirm synergy. Our work does not seek to replace these established applications but rather to optimise how concentrations are selected for such assays. By selecting MIC thresholds defined by bacterial counts, TKCs can be designed to capture both suppression and killing in a balanced and interpretable way, thereby extending the utility of TKCs for pharmacodynamic modelling.

We investigated MIC trailing in *Staphylococcus pseudintermedius* exposed to trimethoprim (TMP) and three sulphonamides, sulfamethoxazole (SMX), sulfadiazine (SDZ) and sulfadimethoxine (SDMX), using broth microdilution according to EUCAST methodology. As inoculum size can impact the likelihood of trailing (Amsterdam 2015; Smith and Kirby 2018) the inoculum size was confirmed by viable count (EUCAST 2024). Given the imprecision of visual MICs for bacteriostatic agents, we hypothesised that a count‐based method would more accurately identify the MIC threshold that limits growth below 10^8^ CFU/mL, the operational definition of MIC based on visible turbidity. This refinement was used to support downstream PD studies, such as TKCs, which aim to include sub‐MIC (0.25×–0.75×) and supra‐MIC (1.5× and above) concentrations to capture partial growth suppression and killing. Accurate identification of the actual (1×) MIC is therefore critical, as it anchors the entire concentration–response relationship and informs the interpretation of pharmacodynamic outcomes (Turnidge and Paterson 2007).

Ten clinical *S. pseudintermedius* isolates were tested against each compound. Isolates were selected to represent typical clinical submissions spanning a range of TMP‐sulphonamide MICs within the susceptible range. None were pre‐selected for resistance traits; the panel was intended to capture natural variability in trailing behaviour under standardised testing conditions. Visual MICs were determined after 24 h based on the lowest concentration showing no visible turbidity, in line with EUCAST guidance. In many cases, TMP and sulphonamides produced diffuse inhibition profiles characterised by reduced turbidity across several wells, without the appearance of a firm inhibitory boundary (EUCAST 2000) leading to inconsistent endpoint calling and misestimation. To address this, we implemented an extension to the standard method by measuring bacterial counts (CFU/mL) at 0 and 24 h for five concentrations spanning the visual MIC. Log_10_ changes in bacterial density were plotted against drug concentration, to define pharmacodynamic thresholds: 0 log_10_ change and +2.3 log_10_ as described in Figure 1. The MIC as a pharmacodynamic target was then defined as the lowest concentration above these thresholds, rounded up to the next dilution step. These thresholds are consistent with previously proposed PK/PD benchmarks (Mouton et al. 2005). It should be noted, however, that the assumption of 10^8^ CFU/mL as the threshold for visible pellet formation is a practical estimate and may vary between bacterial species, depending on cell size, clumping behaviour and growth characteristics. Despite this variability, it remains a useful benchmark for guiding concentration selection for further experiments.

The results (Table 1; Plots in Figures S1–S4) showed that visual MICs frequently underestimated the concentrations required to inhibit growth when trailing was present. For TMP, visual MICs ranged from 1 to 4 μg/mL across strains, but in 5 out of 10 isolates higher concentrations were required to achieve either stasis or prevent growth. For example, isolate 21/0463 had a visual MIC of 4 μg/mL but required 8 μg/mL to reach both the stasis and visible‐growth thresholds. For sulphonamides, discrepancies were more pronounced: visual MICs were often 2‐ to 4‐fold lower than the 2.3 log_10_ inhibition threshold, corresponding to differences of more than one dilution step. Notably, ≥ 2‐dilution discrepancies occurred in only 1/10 (10%) isolates for TMP and 1/10 (10%) for sulfamethoxazole, but in 8/10 (80%) isolates for sulfadiazine and 2/10 (20%) for sulfadimethoxine, indicating that TMP, SMX and SDMX provide more predictable MICs whereas SDZ is especially prone to trailing‐related variability. In several cases, particularly those with pronounced trailing, the concentration required to achieve full stasis or surpass the visible growth threshold lay beyond the highest concentrations tested. To investigate if trailing at the 1:19 trimethoprim to sulphonamide ratio influences MIC determination, three *S. pseudintermedius* isolates were tested. With a focus on the 1:19 ratio because it reflects the conventional fixed‐dose combination used in both human and veterinary medicine and therefore provides the most clinically relevant benchmark for assessing trailing and MIC refinement. Isolates were selected to represent typical clinical isolates with susceptibility to TMP‐sulphonamides. Their TMP/SMX MICs were around the tentative ECOFF (TECOFF) for *Staphylococcus aureus* and below the current EUCAST clinical breakpoint of 2 mg/L (EUCAST 2021). As no MIC distribution, ECOFF or species‐specific breakpoint is currently available for *S. pseudintermedius* , *S. aureus* values were used as the most relevant reference. Visual MICs and log‐change MICs were determined using the same quantitative approach as for single agents (Table 2; Plots in Figure S5).

**TABLE 1:** Comparison of MIC for *S. pseudintermedius* as determined visually and by plotting the relative difference in bacterial growth.

Strain	Trimethoprim			Sulfamethoxazole			Sulfadiazine			Sulfadimethoxine		
	MIC (Count)^a^	MIC (Visual)	Fold‐change^b^ (Count/Visual)	MIC (Count)^a^	MIC (Visual)	Fold‐change^b^ (Count/Visual)	MIC (Count)^a^	MIC (Visual)	Fold‐change^b^ (Count/Visual)	MIC (Count)^a^	MIC (Visual)	Fold‐change^b^ (Count/Visual)
21/0463	8	4	2	128	64	2	128	64	2	64	64	1
23/0024	4	4	1	32	32	1	128	32	4	64	32	2
22/0693	4	4	1	16	8	2	32	8	4	16	8	2
21/0027	4	4	1	16	16	1	128	16	8	32	16	2
21/0443	4	4	1	64	4	16	128	4	16	32	4	8
22/0608	4	2	2	32	16	2	128	16	8	32	16	2
21/0505	4	1	4	16	8	2	64	8	4	32	8	4
22/0285	4	4	1	16	32	0.5	128	32	4	64	32	2
22/0605	4	4	1	16	32	0.5	64	32	2	64	32	2
21/0460	4	4	1	32	16	2	128	16	8	32	16	2

^a^ MIC as determined from the log change in bacterial density (CFU/mL) from an initial inoculum of 5 × 10^5^ CFU/mL rounded up to the nearest AMD concentration. 2.3 Log_10_ represents the visible growth threshold of 10^8^ CFU/mL as described by Mouton et al. (2005).

^b^ Fold‐change values were calculated as the ratio of the MIC determined from bacterial counts (rounded up to the next dilution) to the visually determined MIC for each isolate.

**TABLE 2:** Minimum inhibitory concentration (MIC; mg/L) as determined visually and by assessing the change in bacterial density (CFU/mL) over 24 h for *S. pseudintermedius* against the antimicrobial drugs (AMDs) Trimethoprim in combination with sulfamethoxazole (SMX), sulfadiazine (SDZ) or sulfadimethoxine (SDMX) at a ratio of 1:19 (TMP:Sulphonamide).

AMD	Strain	1:19		
		MIC (Count/Plot)^a^		MIC (Visual)
		2.3 Log_10_	0 Log_10_	
T/SMX	21/0027	0.25	≥ 4	1
	21/0505	0.25	0.5	0.5
	22/0693	0.25	0.5	1
T/SDZ	21/0027	1	2	0.25
	21/0505	1	1	0.5
	22/0693	0.5	1	0.5
T/SDMX	21/0027	0.25	2	1
	21/0505	0.25	0.5	1
	22/0693	0.25	0.5	1

*Note:* MICs reported as the trimethoprim concentration.

^a^ MIC as determined from the log change in bacterial density (CFU/mL) from an initial inoculum of 5 × 10^5^ CFU/mL rounded up to the nearest AMD concentration. 2.3 Log_10_ represents the visible growth threshold of 10^8^ CFU/mL as described by Mouton et al. (2005) and 0 Log_10_ represents stasis (i.e., no change in density).

Across all antifolate compounds, the extended method provided a clearer distinction between active and inactive concentrations. The log_10_ CFU/mL profiles allowed us to pinpoint the inflection between growth suppression and visible‐growth, offering objective, reproducible criteria for MIC designation (Mueller et al. 2004). Hain et al. (2021) utilised a similar approach to define the IC_50_ as the inflection point when investigating sulphonamides alone using pyoverdine fluorescence measurement, our approach provides a straightforward way, using practical measures, to identify the critical endpoints. Table 1 summarises the MICs determined by both methods. The improved resolution may be particularly valuable in isolates exhibiting delayed drug activity where bacterial growth prior to drug action may result in visible turbidity or increased trailing. Time‐kill curves were performed in triplicate for all *Staphylococcus pseudintermedius* isolates with trimethoprim, sulfamethoxazole, sulfadiazine and sulfadimethoxine. Following overnight growth on Mueller–Hinton agar, colonies were suspended in PBS and diluted to a standardised inoculum (~5 × 10^5^ CFU/mL) in cation‐adjusted Mueller–Hinton broth (CAMHB), then incubated for 2 h at 37°C to promote log‐phase growth. TKCs were conducted in 96‐well plates at 0–16× MIC, with timepoints collected over 24 h; plates were incubated statically at 37°C under ambient atmospheric conditions, and inocula were confirmed by viable count. Time‐kill curve data for TMP showed a characteristic lag of up to 1 h before growth suppression became evident, while sulphonamides demonstrated delayed inhibition extending up to 4 h, consistent with slow onset of action or target redundancy in the folate synthesis pathway (Sköld 2000). Figure 4 shows TKCs for TMP and each sulfonamide, with the concentration centralised on the MIC as defined by the 2.3 log_10_ resulting in an equal spread of concentrations that achieve reduced bacterial growth or kill respectively, allowing for better PD assessment of the drug response. Replicate TKCs showed consistent qualitative patterns, with variability in log_10_ CFU/mL generally within ±0.5 across timepoints. This variability is in line with previous reports for in vitro static culture models and did not alter the interpretation of growth suppression or killing dynamics.

In addition to providing descriptive dynamic profiles, TKCs form the basis for PD modelling by quantifying bacterial kill rates across concentrations and time. For combinations, these data allow the assessment of interaction patterns: synergism when the combination achieves greater kill than either drug alone, or antagonism when combined activity is reduced. The present study used TKCs not to confirm the antimicrobial or synergistic effect but to generate concentration‐response data anchored on refined MICs, providing a robust foundation for PD model development and evidencing the applicability of the refined PD MIC for anchoring TKC.

**FIGURE 4:**
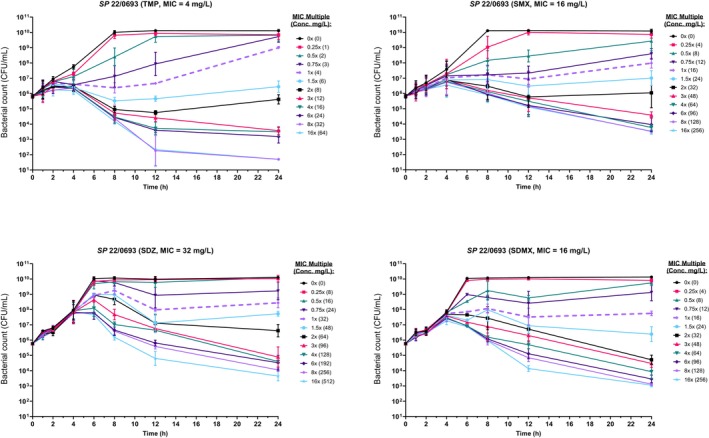
Time‐kill curve (TKCs) for trimethoprim (TMP), sulfamethoxazole (SMX), sulfadiazine (SDZ) and sulfadimethoxine (SDMX) with the MIC described by the 2.3 log_10_ change in bacterial density. Concentration in brackets for each MIC multiple is the true concentration (in mg/L). Centralizing the concentration range around this value results in a focused range of dose‐effect curves for PD analysis.

At the 1:19 TMP:S ratio (Table 2), the visual MICs showed improved alignment with the MICs derived from bacterial density data, particularly the +2.3 log_10_ growth threshold. This closer agreement likely reflects the enhanced bactericidal activity of the synergistic combination, which produced a more abrupt transition from growth to inhibition. As a result, the trailing effect commonly seen with the individual agents was markedly reduced, and a clearer, more visually distinct endpoint was observed. This improved concordance between visual and pharmacodynamic MIC definitions suggests that the 1:19 combination yields more reliably interpretable results for both susceptibility testing and PD study design, as the concentration required to suppress visible growth more closely reflects the concentration that limits bacterial regrowth to below the pellet‐forming threshold. The distinct visual endpoint at 1:19 may therefore offer both practical and mechanistic advantages when characterising the activity of antifolate combinations.

Ambiguity caused by trailing endpoints can be exacerbated under certain conditions. For example, bacteriostatic agents may suppress replication without causing complete inhibition, resulting in partial turbidity. Mueller‐Hinton fastidious (MH‐F) broth, due to its dark colour, can further impair visual detection of subtle turbidity or small pellets, complicating the accurate identification of the MIC threshold. In addition, delayed antimicrobial onset of activity as observed with TMP and sulphonamides, may allow sufficient early bacterial replication to occur before inhibition takes effect, leading to residual growth that can mask the true MIC. Together, these factors represent a ‘perfect storm’ that complicates visual endpoint determination. The approach outlined here, based on quantifying bacterial density, offers clearer, more objective endpoints, reducing variability and improving interpretability both in complex media, across different bacterial species, and for other antimicrobial agents. While this study focused on trimethoprim and sulphonamides in *S. pseudintermedius* , the issue of trailing is not unique to these agents. Similar challenges have been reported for antifungal azoles (Simner and Patel 2020; Zomorodian et al. 2016), and some bacterial species such as Mycobacterium exhibit trailing across a range of antimicrobials, including tetracyclines and aminoglycosides (Park et al. 2023; Shankar et al. 2022). Applying a quantitative refinement based on bacterial (or fungal) density could therefore provide a general strategy to improve MIC assignment and ensure more reliable pharmacodynamic interpretation across a wider range of antimicrobial classes.

These findings have practical implications. In clinical microbiology, MIC misclassification due to trailing may complicate both clinical reporting and PD interpretation; in particular, reliance on visual MICs in trailing‐prone organisms can lead to misclassification in pharmacodynamic contexts. For example, underestimating MICs by one or more dilutions may falsely categorize resistant strains as susceptible, particularly where breakpoint values are near the observed MIC. In pharmacometric applications, reliance on visual MICs in trailing‐prone organisms can produce inaccurate and ‘mis‐centred’ TKC that do not convey the full extent of the kill achievable with this drug and subsequently distort pharmacodynamic indices such as AUC/MIC or T>MIC, leading to inaccurate predictions of efficacy or resistance suppression. The novelty of this study lies not in redefining MIC values, but in showing how a pharmacodynamically guided refinement can repurpose MIC determination as a tool for rational design of time‐kill studies and downstream PD modeling. By minimizing bias from trailing endpoints, this approach ensures that dose–response relationships are properly centered and that PD indices such as AUC/MIC and T>MIC are grounded on concentrations that truly reflect growth suppression rather than ambiguous visual thresholds. Strengthening the microbiological basis of MIC determination in this way improves the reliability of pharmacological analyses and highlights the translational value of microbiological accuracy for pharmacology‐driven dose optimization. Importantly, this method is not intended to replace standard clinical MIC testing, but to provide more precise inputs for PD optimization, thereby supporting more robust modeling and potentially informing PK/PD targets. This density‐based refinement aligns with PD principles and offers mechanistic insight into antimicrobial inhibition.

We recognise that while this quantitative refinement offers clear advantages for pharmacodynamic study design and mechanistic understanding, its routine application in a busy diagnostic laboratory may be limited by the need for additional colony counts and extended analyses. As such, we view this approach primarily as a research tool to support pharmacodynamic investigations and the development of translational models, rather than as a replacement for standardised susceptibility testing in clinical practice. Nonetheless, the principles illustrated here may help inform the refinement of susceptibility breakpoints and improve interpretation where trailing complicates MIC determination. Although the present work is intended as a research tool to support PK/PD modelling rather than ECOFF or breakpoint setting, refinements such as these may still have indirect value for regulators or committees in the future. Specifically, by reducing variability in MIC determination for trailing‐prone agents, this approach could help generate more consistent data to underpin pharmacodynamic analyses that inform dose optimisation. Formal integration into breakpoint‐setting would require coordinated validation across multiple laboratories, which is beyond the scope of our study, but the principles outlined here may nonetheless support the broader dialogue between pharmacologists, microbiologists and regulatory bodies.

In conclusion, visual MIC determination is vulnerable to error in the presence of trailing endpoints, especially for TMP and sulphonamides in *S. pseudintermedius* . A quantitative refinement using log_10_ CFU/mL change across MIC‐adjacent concentrations improves resolution, reproducibility and interpretive accuracy. This approach is well aligned with pharmacodynamic theory and may be particularly useful in supporting translational research and regulatory susceptibility testing for compounds prone to ambiguous inhibition profiles.

## Author Contributions

A.M. and L.P. contributed to the conception and design of the study. A.M. organized and performed all aspects of the study, and analysis. A.M. wrote the first draft of the manuscript. All authors contributed to the manuscript revision, read and approved the submitted version.

## Conflicts of Interest

The authors declare no conflicts of interest.

## Supporting information


**Figure S1:** Plot of log change in bacterial count (CFU/mL) during overnight exposure at 2‐fold increasing concentrations of trimethoprim (T). The dotted line represents the visible growth threshold of 10^8^ CFU/mL as described by Mouton et al. 2005 (i.e., log_2.3_) and where it crosses the *x*‐axis (i.e., Log_0_) represents static concentration (i.e., no change in density).
**Figure S2:** Plot of log change in bacterial count (CFU/mL) during overnight exposure at 2‐fold increasing concentrations of sulfamethoxazole (SMX). The dotted line represents the visible growth threshold of 108 CFU/mL as described by Mouton et al. 2005 (i.e., log2.3) and where it crosses the x‐axis (i.e., Log0) represents static concentration (i.e., no change in density).
**Figure S3:** Plot of log change in bacterial count (CFU/mL) during overnight exposure at 2‐fold increasing concentrations of sulfadiazine (SDZ). The dotted line represents the visible growth threshold of 108 CFU/mL as described by Mouton et al. 2005 (i.e., log2.3) and where it crosses the x‐axis (i.e., Log0) represents static concentration (i.e., no change in density).
**Figure S4:** Plot of log change in bacterial count (CFU/mL) during overnight exposure at 2‐fold increasing concentrations of sulfadimethoxine (SDMX). The dotted line represents the visible growth threshold of 108 CFU/mL as described by Mouton et al. 2005 (i.e., log2.3) and where it crosses the x‐axis (i.e., Log0) represents static concentration (i.e., no change in density).
**Figure S5:** Plot of log change in bacterial count (CFU/mL) during overnight exposure at 2‐fold increasing concentrations of T/SMX, T/SDZ and T/SDMX. The dotted line represents the visible growth threshold of 108 CFU/mL as described by Mouton et al. 2005 (i.e., log2.3) and where it crosses the x‐axis (i.e., Log0) represents static concentration (i.e., no change in density).

## Data Availability

Data generated in this study are included in the manuscript and Supporting Information.
