# Supplementary material for: Time Kill Curve PD Modelling Experiments Are Affected by Trailing MIC Endpoints: Refinement of MIC Determination for S. pseudintermedius
Source: J Vet Pharmacol Ther. 2025 Oct 28;49(2):220–7. doi: 10.1111/jvp.70033 (PMC12968473; doi:10.1111/jvp.70033)
Supplement: Supplementary file 1 — Figure S1: Plot of log change in bacterial count (CFU/mL) during overnight exposure at 2‐fold increasing concentrations of trimethoprim (T). The dotted line represents the visible growth threshold of 108 CFU/mL as described by Mouton et al. 2005 (i.e., log2.3) and where it crosses the x‐axis (i.e., Log0) represents static concentration (i.e., no change in density). Figure S2: Plot of log change in bacterial count (CFU/mL) during overnight exposure at 2‐fold increasing concentrations of sulfamethoxazole (SMX). The dotted line represents the visible growth threshold of 108 CFU/mL as described by Mouton et al. 2005 (i.e., log2.3) and where it crosses the x‐axis (i.e., Log0) represents static concentration (i.e., no change in density). Figure S3: Plot of log change in bacterial count (CFU/mL) during overnight exposure at 2‐fold increasing concentrations of sulfadiazine (SDZ). The dotted line represents the visible growth threshold of 108 CFU/mL as described by Mouton et al. 2005 (i.e., log2.3) and where it crosses the x‐axis (i.e., Log0) represents static concentration (i.e., no change in density). Figure S4: Plot of log change in bacterial count (CFU/mL) during overnight exposure at 2‐fold increasing concentrations of sulfadimethoxine (SDMX). The dotted line represents the visible growth threshold of 108 CFU/mL as described by Mouton et al. 2005 (i.e., log2.3) and where it crosses the x‐axis (i.e., Log0) represents static concentration (i.e., no change in density). Figure S5: Plot of log change in bacterial count (CFU/mL) during overnight exposure at 2‐fold increasing concentrations of T/SMX, T/SDZ and T/SDMX. The dotted line represents the visible growth threshold of 108 CFU/mL as described by Mouton et al. 2005 (i.e., log2.3) and where it crosses the x‐axis (i.e., Log0) represents static concentration (i.e., no change in density). [file JVP-49-220-s001.docx]

Supplementary Figure 1: Plot of log change in bacterial count (CFU/mL) during overnight exposure at 2-fold increasing concentrations of trimethoprim (T). The dotted line represents the visible growth threshold of 10^8^ CFU/mL as described by Mouton et al. 2005 (i.e. log2.3) and where it crosses the x-axis (i.e. Log0) represents static concentration (i.e. no change in density).

Supplementary Figure 2: Plot of log change in bacterial count (CFU/mL) during overnight exposure at 2-fold increasing concentrations of sulfamethoxazole (SMX). The dotted line represents the visible growth threshold of 10^8^ CFU/mL as described by Mouton et al. 2005 (i.e. log2.3) and where it crosses the x-axis (i.e. Log0) represents static concentration (i.e. no change in density).

Supplementary Figure 3: Plot of log change in bacterial count (CFU/mL) during overnight exposure at 2-fold increasing concentrations of sulfadiazine (SDZ). The dotted line represents the visible growth threshold of 10^8^ CFU/mL as described by Mouton et al. 2005 (i.e. log2.3) and where it crosses the x-axis (i.e. Log0) represents static concentration (i.e. no change in density).

Supplementary Figure 4: Plot of log change in bacterial count (CFU/mL) during overnight exposure at 2-fold increasing concentrations of sulfadimethoxine (SDMX). The dotted line represents the visible growth threshold of 10^8^ CFU/mL as described by Mouton et al. 2005 (i.e. log2.3) and where it crosses the x-axis (i.e. Log0) represents static concentration (i.e. no change in density).

Supplementary Figure 5: Plot of log change in bacterial count (CFU/mL) during overnight exposure at 2-fold increasing concentrations of T/SMX, T/SDZ, and T/SDMX. The dotted line represents the visible growth threshold of 10^8^ CFU/mL as described by Mouton et al. 2005 (i.e. log2.3) and where it crosses the x-axis (i.e. Log0) represents static concentration (i.e. no change in density).
